# A tremella-like *in situ* synthesis of ZIF-67Co(OH)F@Co_3_O_4_ on carbon cloth as an electrode material for supercapacitors†

**DOI:** 10.1039/d4ra04250f

**Published:** 2024-09-04

**Authors:** Shakeel Ahmad, Muhammad Tariq, Zia Ur Rehman, Shanshan Yao, Bing Zhu, Henmei Ni, Muhammad Samiuddin, Khalid Ali Khan, Magdi E. A. Zaki

**Affiliations:** a School of Chemistry and Chemical Engineering, Southeast University Nanjing 211189 PR China henmei_ni@seu.edu.cn; b Institute for Advanced Materials, College of Materials Science and Engineering, Jiangsu University Zhenjiang 212013 P. R. China; c Metallurgical Engineering Department, NED University of Engineering and Technology Karachi 75850 Pakistan; d Applied College, Center of Bee Research and its Products (CBRP), Unit of Bee Research and Honey Production, King Khalid University P.O. Box 9004 Abha 61413 Saudi Arabia; e Department of Chemistry, College of Science, Imam Mohammad Ibn Saud Islamic University Riyadh 11623 Saudi Arabia

## Abstract

In this study, a simple *in situ* technique followed by hydrothermal method is used to synthesize a novel tremella-like structure of ZIF-67Co(OH)F@Co_3_O_4_/CC metal–organic framework (MOF) derived from zeolite imidazole. The *in situ* synthesis of metal–organic frameworks (MOFs) increases their conductivity and produces more active sites for ion insertion. Their unique, scalable design not only provides more space to accommodate volume change but also facilitates electrolyte penetration into the electrode resulting in more active materials being utilized and ion-electron transfer occurring faster during the cycle. As a result, the binder-free ZIF-67Co(OH)F@Co_3_O_4_/CC supercapacitor electrode exhibits typical pseudo-capacitance behaviour, with a specific capacitance of 442 F g^−1^ and excellent long-term cycling stability of 90% after 5000 cycles at 10 A g^−1^.

## Abstract 2

In this study, a simple *in situ* technique followed by hydrothermal method is used to synthesize a novel tremella-like structure of ZIF-67Co(OH)F@Co_3_O_4_/CC metal–organic framework (MOF) derived from zeolite imidazole.

## Graphical abstract

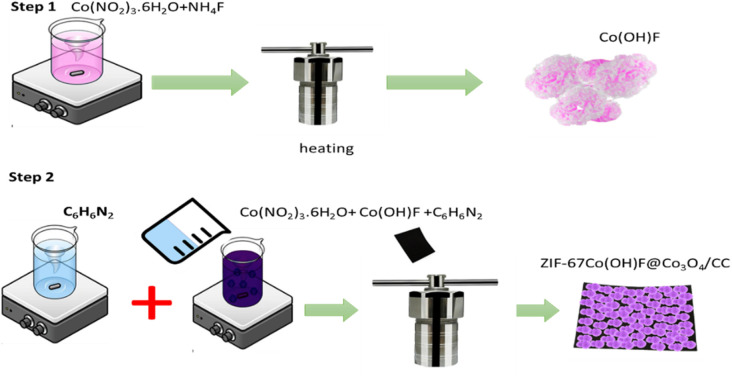

## 1. Introduction

Rapid global economic development has led to the depletion of non-renewable energy resources, prompting researchers to explore technological advancements to meet the growing demand for efficient and renewable energy. Supercapacitors have emerged as a highly promising solution owing to their fast charge/discharge rate, excellent cyclic stability, and high power density.^1^ Various nanomaterials have been established to improve electrochemical properties. Among these electrode materials, carbon-based materials, including porous carbons (PC)^2–6^ graphene and carbon nanotubes have gained significant recognition for their cost-effectiveness, high specific capacitance, and environmentally friendly characteristics.^7–12^ Thermal decomposition of metal–organic frameworks (MOFs) to produce porous carbon materials has attracted significant attention owing to their unique structures, large specific surface areas, and abundant pore structures.^13^ Typically, MOFs are constructed using central metals from d and f-block elements, such as Zn, Co, Zr, *etc.*^14,15^ Although tunable pore size and high surface area are advantageous for electrochemical applications of MOFs, however, their poor electrical conductivity and electroactivity limit their performance. To address these limitations, various post-treatments have been employed, including carbonization, sulfurization, oxidation, salinization, and fluorination.^16^ Binder-free electrodes, which eliminate the need for additional binders to adhere the active material onto a conductive substrate show promise in preventing poor adhesion and can be fabricated using techniques such as doctor-blade or dip-coating.^17^ Researchers have explored different approaches to improve the electrical conductivity and electroactivity of MOFs. For example, Huang *et al.* fused a carbonized zeolitic imidazolate (ZIF-67) framework on nickel foam to make a binder-free battery-like electrode.^18^ Wu *et al.* studied the effect of carbonization temperature on the energy storage capabilities of UiO-66-derived carbon.^19^ Cheng *et al.* make sulfurized ZIF-67 binder-free electrodes on nickel foam for BSH battery-type electrode.^20^ Xiao *et al.* used different sulfur sources to synthesize Co-MOF binder-free BSH electrode.^21^ Wu *et al.* synthesized MIL 101 derivative by carbonization oxidation process for electrochemical performance.^22^ They also synthesized ZFI-67 derivatives with ammonium fluoride and salinization process as electroactive materials for BSH.^23^ Wang *et al.* utilized a facile one-step solution process to fabricate ammonia borane fluoride-induced ZIF-67 derivatives for energy storage.^24^ Liu *et al.* synthesized a honeycomb-like porous carbon mettle organic framework derived from fluorinated magnesium as electrode materials for supercapacitors.^25^ Although post-treatments have shown promise, which involve additional steps that may increase time and cost. Moreover, the replacement of expensive ligands with post-treated anions might limit the full utilization of ligands. Therefore, *in situ* modification provides an alternative approach to enhance the electrical conductivity and electroactivity of MOFs, as compared to *ex situ* post-treatments^26,27^ Co_3_O_4_ is considered to be an ideal electrode material for supercapacitors due to its low-cost, high theoretical capacitance (3600 F g^−1^) and high electrochemical stability.^28^ However, in practical use during the reaction process reduces its electrochemical performance due to slow electron transfer rate and agglomeration of Co_3_O_4_.^29^ It is necessary to synthesize electrode materials that are short in the electron transport channel, having fast reaction rate, and have good stability. Strong structural stability, high porosity, and a large specific surface area make ZIF-67 material an excellent precursor for the synthesis of transition metal oxides (TMOs).^30^ Many research work on the use of ZIF-67 as a precursor in the synthesis of cobalt oxide have been published by late Sun *et al.* used ZIF-67 as a precursor and direct annealing in air at various temperatures to obtain Co_3_O_4_ nanoparticles. The results demonstrate that temperature has a significant impact on the composition and characteristics of Co_3_O_4_ nanoparticles.^31^ Carbon nanotubes (CNTs) were *in situ* implanted by Qu *et al.*^32^ into the porous Co_3_O_4_ dodecahedron that was derived from ZIF-67. The composites' morphology mostly preserved the dodecahedron structure. The composites, shape, and electrical conductivity were improved by controlling the amount of carbon nanotubes. Because of its numerous redox reactions. Cobalt hydroxide (Co(OH)_2_) is another cobalt molecule that has been thoroughly researched. In redox processes, cobalt hydroxide contributes more electrons than nickel oxide and hydroxide. As a result, cobalt hydroxide has a theoretically higher specific capacitance than nickel oxide and hydroxide.^33^ For example, Zhang *et al.* synthesized Co(OH)F nano rods by hydrothermal method they examined the electrochemical performance for super capacitor which give outstanding value of 1265 mF cm^−2^.^34^ Liu *et al.* prepared Co(OH)F/Ni(OH)_2_ hybrid structure which possess 104 C g^−1^ specific capacitance with 94% stability.^35^ Wang *et al.* developed aluminum doped cobalt hydroxide fluoride nano sheets which show the maximum specific capacitance of 1576 F g^−1^ at 1 A g^−1^.^36^ John and it group synthesized cobalt fluoride hydroxide hybrid super capacitor which possess 389 C g^−1^ specific capacitance.^37^ Mei *et al.* prepared Co_3_O_4/_Co(OH)_2_ 3D nano network by two step hydrothermal method which possess 876 F g^−1^ specific capacitance at 2 A g^−1^ current density^31^ Pang *et al.* prepared Co_3_O_4_ nanocube/Co(OH)_2_^−^ by one step controllable hydrothermal method which show large specific capacitance value of 1164 F g^−1^ at 1.2 A g^−1^.^32^

In this work, we adjusted the temperature for the synthesis of nanocomposites, to synthesize a novel binder-free electrode ZIF-67Co(OH)F@Co_3_O_4_ for supercapacitors. A precursor solution containing Co(OH)F nanoparticles indicates to the formation of a metal hydroxide, fluoride, zeolite imidazole framework. Using a simple *in situ* technique followed by hydrothermal procedure, we synthesized the typical ZIF-67 metal–organic framework (MOF), which has a tremella-like structure with a high surface area. ZIF-67 Co(OH)F@Co_3_O_4_ nanocomposite are fused to carbon fabric to form the ZIF-67Co(OH)F@Co_3_O_4_/CC supercapacitor electrode. The advantages of both a conductive substrate and the ZIF-67 Co(OH)F@Co_3_O_4_ are combined in this hybrid structure. The composite not only provides a large number of electroactive sites for effective redox reactions, but also accelerating ion transport and electrolyte diffusion. With the combined benefits of the one-dimensional conductive carbon fibers and the large surface area of tremella-type ZIF-67Co(OH)F@Co_3_O_4_ MOF, electrode has a specific capacitance of 442 F g^−1^. The electrode continues to retain 90% of its stability after 5000 cycles, indicating remarkable long-term cyclic stability.

## 2. Experimental

### 2.1 Chemicals

The chemicals used in this study were purchased from Aladdin bio chemical technology Shanghai China. The chemicals procured from the supplier included Co(No_3_)_2_·6H_2_0, ammonium fluoride, 2-methylimidazole, methanol, ethanol, and potassium hydroxide. These chemicals were selected based on their suitability for the synthesis and characterization of the desired cobaltZIF-67 (CoOH)F@Co_3_O_4_ nanocomposites. A Co(No_3_)_2_·6H_2_0, was employed as a cobalt precursor for the synthesis of cobalt based MOF. Ammonium fluoride was used as a source of fluoride ions, while 2-methylimidazole served as a ligand in the formation of the metal organic zeolite imidazole framework. Methanol and ethanol were utilized as solvents for the reactions, providing a suitable medium for the chemical processes.

### 2.2 Synthesis of Co(OH)F nanoparticles

Co(OH)F nanoparticles was synthesized by adding 1 g Co(NO_2_)_3_·6H_2_O to 80 mL ethanol followed by addition of 0.5 g of ammonium and stirred for 40 minutes. After 40 minutes of stirring the solution was added to a Teflon line autoclave and heated for 4 h at 120 °C. On completion of reaction, the product was allowed to cool at room temperature, the product was centrifuged, washed several times with water and ethanol. Finally, the product was dried overnight at 70 °C in an oven for further use.

### 2.3 *In situ*, construction of ZiF-67 derived Co(OH)F @Co_3_O_4_/CC electrode

**Fig. 1:**
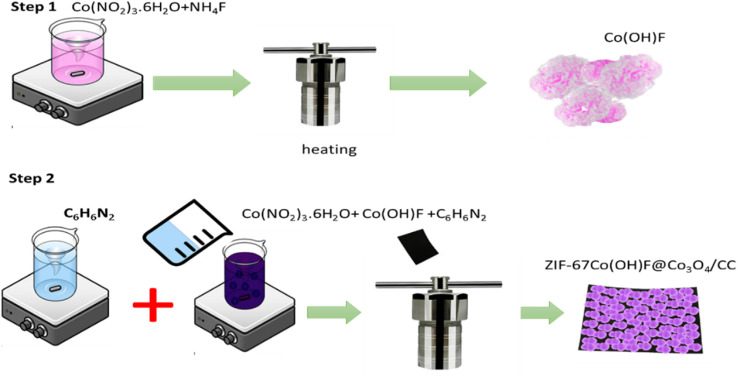
Schematic diagram of ZIF-67Co(OH)F@Co_3_O_4_/CC synthesis.

ZIF-67 was prepared by previously reported method with a little modification.^38^ Both Co(NO_2_)_3_·6H_2_O (0.5 g) and 2-methyl imidazole (1.3 g) were dissolved separately in two beakers already containing 40 mL methanol and stirred for 15 minutes. On complete dissolution, 2-methyl imidazole solution was added to Co(NO_2_)_3_·6H_2_O solution drop wise and then 0.8 g of synthesized Co(OH)F nanoparticles were added and stirred for 1 h. After 1 h stirring the solution was transferred to a Teflon line autoclave and a piece of carbon fiber cloth of 1 × 2 cm^2^ diameter immersed vertically in the solution mixture and keep at different temperature for 5 h, after the desired reaction time, the auto clave reactor was slowly cool down to room temperature the ZIF-67Co(OH)F@Co_3_O_4_ nano composites grow on the surface of carbon cloth after being kept at different temperature for 5 h. The carbon cloth washed for several time with water and ethanol and dry at 80 °C. The fabrication procedure of ZIF-67Co(OHOF@Co_3_O_4_/CC is shown in the Fig. 1. The products synthesized by different temperature given name Co(OHO)F/CC120, Co(OHO)F@Co_3_O_4_/CC160, and Co(OHO)F@Co_3_O_4_/CC200.

### 2.4 Electrochemical analysis

Three electrode systems were made in 1 M KOH electrolyte, with the ZIF-67Co(OH)F@Co_3_O_4_/CC200 serving as the working electrode. Hg/HgO is used as a reference electrode, while platinum foil serves as a counter electrode. The electrochemical measurements were performed using the CHI660E, electrochemical workstation, which included cyclic voltammetry (CV), electrochemical impedance spectroscopy (EIS), and galvanostatic charge discharge (GCD). Eqn (1) is used to get the *C*_F_ value from the GCD curve where *I* is the current density (A g^−1^), Δ*t* is the discharge duration, and *m* is the mass of the active materials. Eqn (2) and (3) are used to calculate the energy density (*E*) and power density (*P*) respectively d*v* is the potential window (*V*), d*t* is the discharge duration (s) and *C* is specific capacitance.^34^1
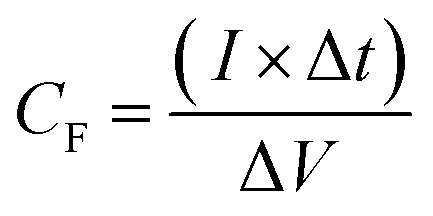
2
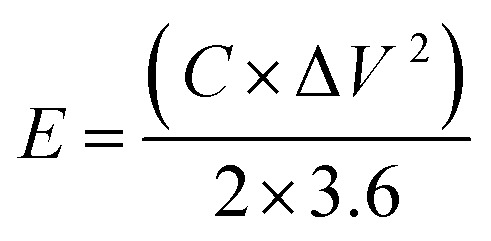
3
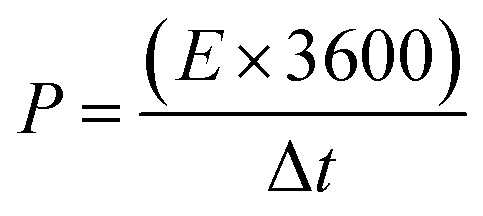


### 2.5. Assembly of the ZIF-67Co(OH)F@Co_3_O_4_/CC//AC flexible supercapacitor

A standard procedure was followed in the fabrication of the negative electrode.^39^*N*-Methyl 2-pyrrolidane was combined with acetylene black and activated carbon (AC) at a mass ratio of 1 : 1 : 8 to create a homogeneous slurry. The current collector carbon cloth electrode was immersed in the slurry for 2 minutes and then dried for 10 h at 80 °C. Gel electrolyte was used in the assembling of ZIF-67Co(OH)F@Co_3_O_4_/CC//AC device. 1 g of PVDF was gradually added to a 10 mL 1 M KOH solution while being continuously stirring at room temperature until the mixture turned transparent. Both the positive and negative electrode were submerged in the gel electrolyte and was held for 30 s. A 1 × 1 cm^2^ of filter paper was used as a separator between the electrodes.

## 3. Results and discussion

### 3.1. X-ray diffraction analysis

**Fig. 2:**
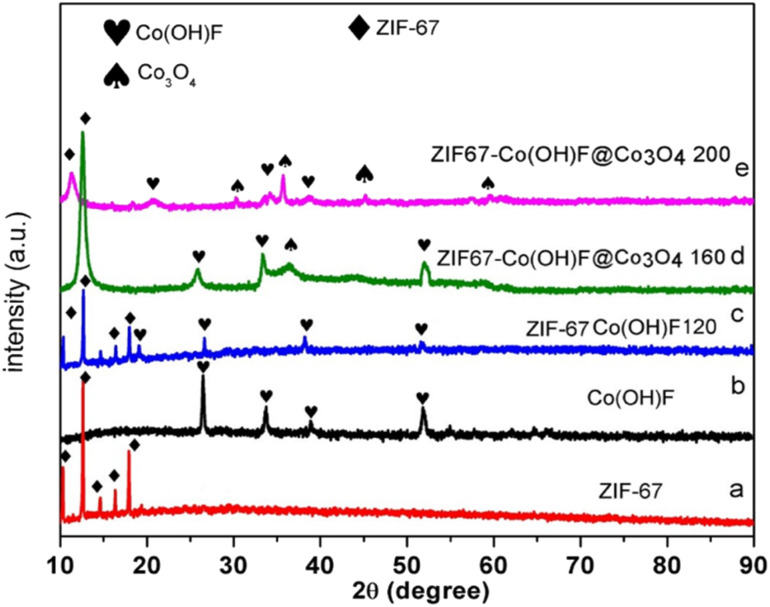
The XRD pattern of ZIF-67 (a) ZIF-67 derived Co(OH)F, (b) (c–e) ZIF-67Co(OH)F@Co_3_O_4_/CC120, 160, and 200 nanocomposites.

Powdered X-rays diffraction (XRD) was used to study the phase purity and crystal structure of the synthesized samples shown in Fig. 2. Fig. 2a displays the XRD pattern of ZIF-67, indicating the relative intensity and peak positions which is reliable according to the literature at 10°, 12.30°, 13°, 16° and 18°.^33^Fig. 2b shows the XRD pattern of Co(OH)F, the reflection peaks at 26.50°, 33.70°, 38.23° and 51.41° for the (003), (201), (211), and (221) planes of Co(OH)F according to [JCPDS No. 50-0827].^40^ XRD pattern of ZIF-67Co(OH)F120 is shown in Fig. 2c the main diffraction peaks at 26°, 35°, 38° and 51° are attributed to (003) (311), (201),and (211) reflection planes of Co(OH)F according to [JCPDS No. 50-0827].^38^ The XRD result of the product synthesized at 120 °C propose the presence of cobalt hydroxide fluoride in the sample. Typical diffraction peaks of ZIF-67 which are present at 10°, 12°, 15°, 17° and 18° which is reliable according to the literature.^41^ For the confirmation of the successful synthesis of ZIF-67Co(OH)F@Co_3_O_4_ the XRD pattern of the samples obtained from ZIF-67Co(OH)F@Co_3_O_4_160, and ZIF-67Co(OH)F@Co_3_O_4_200. The XRD pattern of ZIF-67Co(OH)F@Co_3_O_4_ 160, shows in Fig. 2d the main diffraction peaks at 26°,33°, 38°, and 51° are attributed to (110), (311), (201), and (211) planes of Co(OH)F according to [JCPDS No. 50-0827].^42^ Moreover, the peaks at 19° and 36° attributed (003) and (311) are detected for Co_3_O_4_ according to [JCPDS No. 43-1003].^43^ It is confirmed some of the Co(OH)F are converted to Co_3_O_4_. The XRD pattern of ZIF-67Co(OH)F@Co_3_O_4_200 is shows in Fig. 2e, there are four main diffraction peaks at 31.2°, 36°, 44°, and 59.56° are attributed to (220), (331), (440), and (511) planes of Co_3_O_4_ according to [JCPDS No. 43-1003].^44^ Moreover, the peaks at 20°, 33°, and 38.32° are indexed to the crystalline planes of Co(OH)F according to [JCPDS No. 50-0827] it is confirmed the Co(OH)F are still present in the synthesized composite. Although the diffraction peak of ZIF-67 in ZIF-67Co(OH)F@Co_3_O_4_200 becomes weak but still exists at 11.12°, the peaks intensity of ZIF-67 become low it is due to phase conversion from Co(OH)F to Co_3_O_4_.

### 3.2. Scanning electron microscopy analysis

**Fig. 3:**
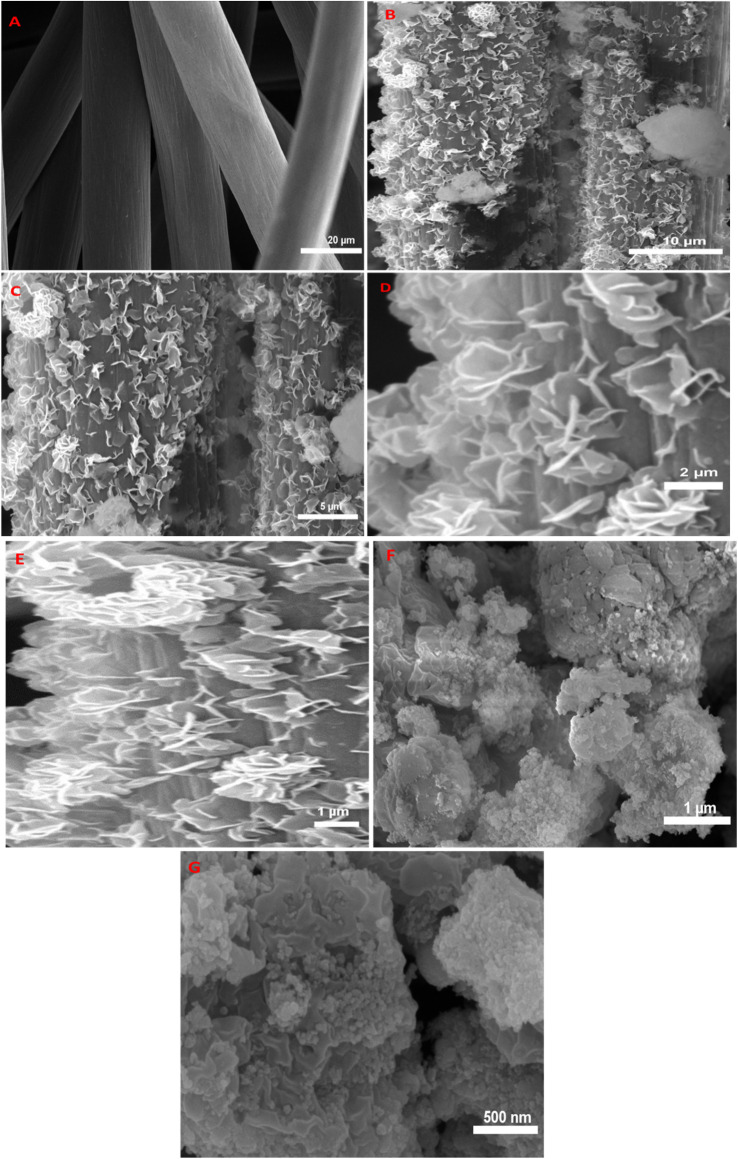
SEM images of (A) carbon cloth, (B to E) ZIF-67 Co(OH)F@Co_3_O_4_/CC200 and (F and G) Co(OH)F.

The morphologies of the ZIF-67Co(OH)F@Co_3_O_4_/CC synthesized at different temperatures were observed by scanning electron microcopy (SEM). The SEM images of carbon cloth before and after the attachment of ZIF-67Co(OH)F@Co_3_O_4_ are shown in the Fig. 3. As shown in the Fig. 3A, the surface of carbon cloth with a diameter of 20 μm, the image indicates that the carbon cloth is smooth, and the individual fibers are well separated from each other. Fig. 3B–E depict the attachment of ZIF-67Co(OH)F@Co_3_O_4_ to carbon cloth. After the combination of ZIF-67Co(OH)F@Co_3_O_4_200 it is confirmed from the SEM images the tremella-like nanosheets are uniformly organized on the surface of carbon cloth. Tremella-like ZIF-67Co(OH)F@Co_3_O_4_200 have superior structure stability, and exhibit greater electrochemical performance which facilitates ion insertion and extraction.^45^Fig. 3F and G shows the SEM images of Co(OH)F which exhibit nano sheets like structure. SEM images of ZIF-67Co(OH)F 120/CC and ZIF-67Co(OH)F@Co_3_O_4_ 160/CC are shown in Fig. S1 and S2 in ESI.† ZIF-67Co(OH)F@Co_3_O_4_ 160/CC possesses the nano sheets-like morphology which are uniformly spread on the surface of carbon cloth while the ZIF-67Co(OH)F120/CC retained square like structure. In summary, it found the novel tremella-like structure can provide a large specific surface area, which causes the collection of more charge in redox reactions the results improved electrochemical performance. Furthermore, energy dispersive spectroscopy (EDS) spectrum of ZIF-67Co(OH)F@Co_3_O_4_/CC200 is shown in Fig. S3 (ESI†). It confirms the presence of carbon (C), cobalt (Co), oxygen (O) and fluorine (F) elements spread over the carbon cloth. Notably, the Co, O and F are spread throughout the entire fiber's cloth. These results validate the successful attachment of ZIF-67Co(OH)F@Co_3_O_4_ nano sheets onto the surface of carbon cloth substrate.

**Fig. 4:**
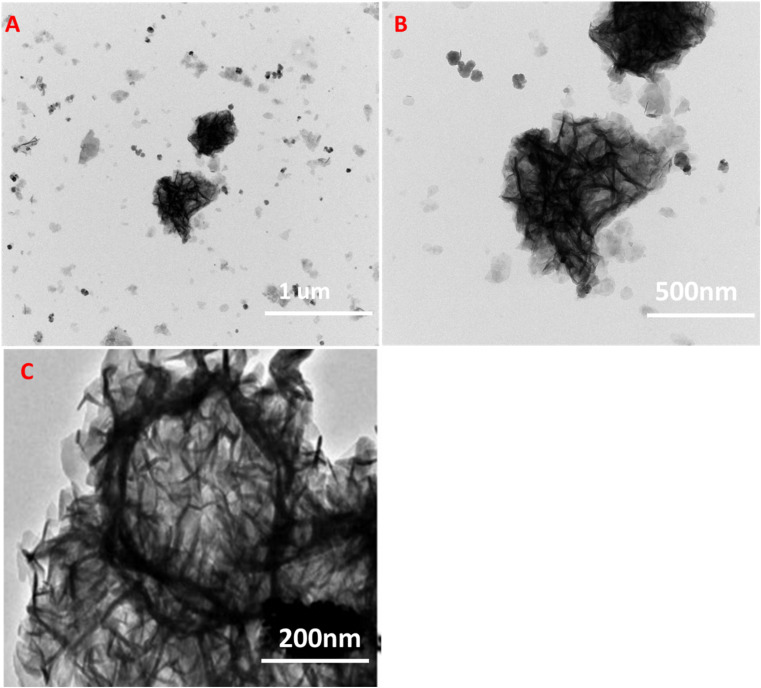
(A and B)TEM images of ZIF-67 Co(OH)F@Co_3_O_4_200 of low magnification (C) TEM images of ZIF-67 Co(OH)F@Co_3_O_4_200 of high magnification.

Further to investigate the composites structure the TEM analysis was carried out. Fig. 4(A–C) shows the TEM images of ZIF-67 Co(OH)F@Co_2_O_4_200 it is observed that the micro structure of ZIF-67 Co(OH)F@Co_2_O_4_200 exhibits the tremella-like structure assembled from leaf-like nanosheets it should be noted that these nanosheets are obviously thinner Among the four structures, it is clear that the nanosheet assembled tremella-like structure is superior to the other structures, because it can provide a richer surface area for the ion-accessible accommodation and promote the ion diffusion, which is advantageous for the ion/extraction processes and the redox reaction at ZiF-67 Co(OH)F@Co_2_O_4_200 the electrode/electrolyte interface.

### 3.3. X-ray photoelectron spectroscopy analysis

**Fig. 5:**
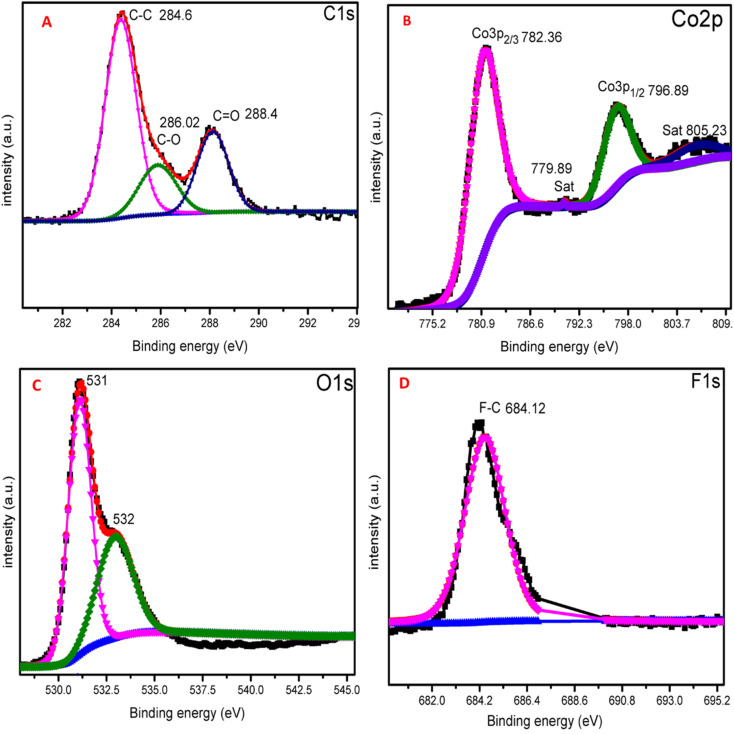
XPS scans of ZIF-67 Co(OH)F@Co_3_O_4_ (A) C 1s, (B), Co 2p, (C) O 1s and (D) F 1s.

X-ray photoelectron spectroscopy (XPS) investigation was carried out to better understand the chemical state of elements in ZIF-67 Co(OH)F@Co_3_O_4_200. Fig. 5 shows the scan spectrum, which indicate the elements C, Co, F, and O. Fig. 5A display the high resolution C 1s spectrum which include the peaks at binding energy 284 eV, 286 eV and 288 eV, assigned to C=C, C–C and C=O.^46,47^Fig. 5B shows the high spectrum of cobalt element. It can be seen that the spectrum consists of two peaks located at 782 eV and 796 eV corresponding to the electronic state of Co 2p_2/3_ and Co 2p_1/2_ respectively. Moreover, two satellite peaks are also noticed present at 789 eV and 805 eV corresponding to the Co 2p_3/2_ and Co 2p_1/2_ respectively.^48^Fig. 5C show high resolution XPS spectrum of O 1s, the peaks at 531 eV and 532 eV corresponds to the Co–O and C–O bonds respectively.^49^ The high-resolution spectrum of F 1s is shows in Fig. 5D which shows a peak at 684 eV corresponding to the C–F bond.^50^

### 3.4. BET analysis

**Fig. 6:**
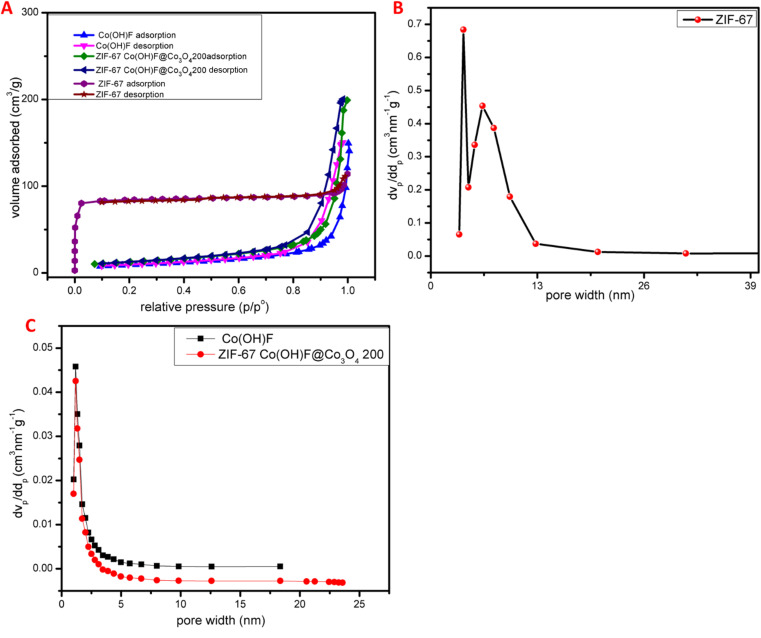
(A) Show the adsorption–desorption isotherm of ZIF-67, Co(OH)F and ZIF-67Co(OH)F@Co_3_O_4_200, (B) pore size distribution of ZIF-67 (C) pore size distribution of Co(OH)F and ZIF-67Co(OH)F@Co_3_O_4_200.

The porosity and surface area of ZIF-67 were characterized by BET analysis the isotherm of ZIF-67 is shown in Fig. 6A which show type I-pattern the adsorption–desorption isotherms showed a steep rise at low relative pressure and then quickly attained a balance to suggest dominant microporous characteristic. The values of specific surface area for ZIF-67 were estimated to be 295 m^2^ g^−1^ the Barrett–Joyner–Halenda pore size distribution plots for the samples of ZIF-67 is shown in Fig. 6B the diameter of most of the pores was found to lie within the range of 1–40 nm. The values of mean centered porosities were assessed as 1.26 and 1.73 nm, respectively. Also, nitrogen gas adsorption–desorption isotherm was used to estimate the porosity and specific surface area of Co(OH)F and ZIF-67Co(OH)F@Co_3_O_4_200 nanocomposites. The isotherm of the Co(OH)F and ZIF-67Co(OH)F@Co_3_O_4_200 as shown in Fig. 6A exists similar to type II pattern, with molten layer development occurring at medium pressure and capillary condensation occurring at high pressure, the flat middle zone represents a monolayer formation. The specific surface area for Co(OH)F and ZIF-67Co(OH)F@Co_3_O_4_200 is 370 m^2^ g^−1^ and 547 m^2^ g^−1^ based on BET, analysis. Barrett–Joyner Halenda (BJH) pore size distribution curve of Co(OH)F and ZIF-67Co(OH)F@Co_3_O_4_200 is shown in Fig. 6C representing a huge number of pores present in the 0–10 nm region. The average pore volume and pore diameter for Co(OH)F and ZIF-67Co(OH)F@Co_3_O_4_200 is 2.8 cm^3^ g^−1^, 4 cm^3^ g^−1^ and 19.14 nm, 25.6619 nm respectively. Which confirms that Co(OH)F@Co_3_O_4_ has greater surface area and provide more active site for electrochemical reaction and enhance the overall performance.

### 3.5. Electrochemical performance

**Fig. 7:**
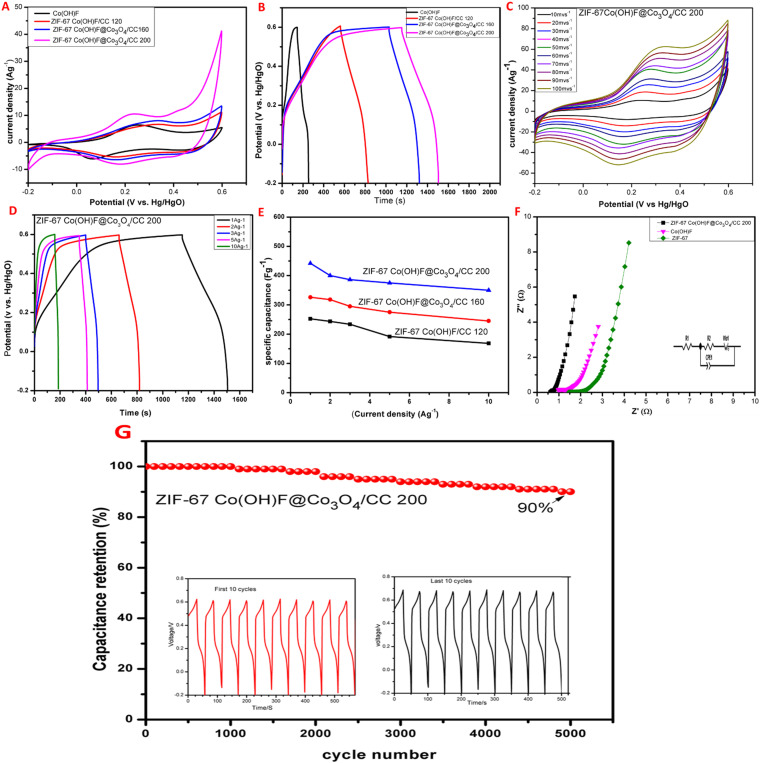
Electrochemical performance of Co(OH)F, ZIF-67Co(OH)F@Co_3_O_4_/CC electrodes, (A) CV curve of Co(OH)F, ZIF-67Co(OH)F@Co_3_O_4_/CC200, 160 and 120 electrodes at 10 mV s^−1^, (B) GCD curves ofCO(OH)F, ZIF-67Co(OH)F@Co_3_O_4_/CC200, 160 and 120 electrodes at 1 A g^−1^, (C) CV curve of ZIF-67Co(OH)F@Co_3_O_4_/CC200 electrode (D) GCD curve of ZIF-67Co(OH)F@Co_3_O_4_/CC200 electrode 1, 2, 3, 5, and 10 A g^−1^, (E) specific capacitance *versus* charge–discharge current density of ZIF-67Co(OH)F@Co_3_O_4_/CC200, 160 and 120 electrodes (F) Nyquist plot of ZIF-67, Co(OH)F, and ZIF-67 Co(OH)F@Co_3_O_4_/CC200 electrode, (G) stability at current density of 10 A g^−1^ of ZIF-67Co(OH)F@Co_3_O_4_/CC200 electrode.

The electrochemical performance of Co(OH)F, ZIF-67 Co(OH)F@Co_3_O_4_/CC200, 160 and 120 electrodes were tested in 1 M KOH electrolyte shown in Fig. 7. The CV curves of all the samples are measured at the same scan rate of 10 mV s^−1^, it is seen that all the CV curves have a pair of redox peaks, confirming the Faraday reaction has occurred shown in Fig. 7A.^51^ Meanwhile, the CV curves area of ZIF-67 Co(OH)F@Co_3_O_4_/CC200 is significantly higher than the other which shows that the ZIF-67Co(OH)F@Co_3_O_4_/CC200 has high utilization of the active site, greater capacity and good electrochemical performance due to its high surface area confirming the results are reliable with SEM images. Fig. 7B shows the GCD curves of all the samples ZIF-67 Co(OH)F@Co_3_O_4_/CC200, 160, and 120 and Co(OH)F at 1 A g^−1^ with specific capacitance value, 442 F g^−1^, 371 F g^−1^, 345 F g^−1^ and 165 F g^−1^ respectively, corresponding to capacity 98 mA h g^−1^,81 mA h g^−1^, 74 mA h g^−1^ and 34 mA h g^−1^. It can be seen that the discharge time of ZIF-67 Co(OH)F@Co_3_O_4_/CC200 is higher than the other. The CV curves of ZIF-67Co(OH)F@Co_3_O_4_200 at scan rate of 1 mV s^−1^ to 100 mV s^−1^ with a potential window of −0.2 V to 0.6 V are displayed in Fig. 7C. With increase in the scan rate there is a sharp increase in the area and the shape remain similar with obvious redox peaks, which shows excellent performance. The redox reaction mechanism shows in the following eqn (4) and (5).^52,53^ The CV curves of ZIF-67 Co(OH)F@Co_3_O_4_/CC 160 and ZIF-67 Co(OH)F/CC 120 are shown in Fig. S4 in the ESI† for comparison all the CV curves show good redox peaks.

4 Co_3_O_4_ + OH^−^ + H_2_O ↔ 3CoOOH + e^−^

5 CoOOH + OH^−^ ↔ CoO_2_ + H_2_O + e^−^

The GCD curves of ZIF-67Co(OH)F@Co_3_O_4_/CC200 is shown in Fig. 7D at current density ranging from 1 A g^−1^ to 10 A g^−1^ with *C*_F_ 442 F g^−1^, 436 F g^−1^, 420 F g^−1^, 386 F g^−1^ and 350 F g^−1^ corresponding to capacity 98 mA h g^−1^, 91 mA h g^−1^, 85 mA h g^−1^, 79 mA h g^−1^, and 69 mA h g^−1^ respectively. The GCD curves of Co(OH)F, ZIF-67-Co(OH)F@Co_3_O_4_/CC160, ZIF-67Co(OH)F/CC120 is shown in Fig (S5) in ESI† for comparison. The GCD curves of all the samples are nonlinear and well symmetrical at all current densities. The specific capacitance of all the samples ZIF-67Co(OH)F@Co_3_O_4_/CC200, 160 and 120 were calculated according to the discharge curves shows in Fig. 7E. The values of specific capacitance decreases with increase the current density this is because at high current density less ion can reach the electrode surface.^54^ The specific capacitance of ZIF-67 Co(OH)F@Co_3_O_4_200 is higher than the other due to its high surface area. Nyquist plot of ZIF-67, Co(OH)F and ZIF-67Co(OH)F@Co_3_O_4_/CC200 were investigated in the frequency range of 0.01 to 1000 kHz. The EIS measurement, Nyquist plots and their fitting are shown in Fig. 7F at high frequency, the intersection with the *X*-axis suggests the equivalent series resistances (*R*_s_), which represent the total resistance in the electrochemical system. Charge transfer resistance (*R*_ct_) and constant phase element (CPE) which represent the electrical resistances at the electrode and electrolyte interface. *W*_0_ (Warburg element) is used as a diffusion resistance to reach the diffusion of the electrolyte within the electrode. Based on fitting results. ZIF-67 and Co(OH)F has a relatively less conductivity in its native form. After the incorporation of Co(OH)F@CO_3_O_4_ the ZIF-67 Co(OH)F@CO_3_O_4_ was characterized with lower values of *R*_s_ and *R*_ct_ (*R*_s_ = 0.55 Ω and *R*_ct_ = 0.104 Ω) than the pristine ZIF-67 and Co(OH)F electrodes (*R*_s_ = 1.38 Ω and *R*_ct_ = 1.04 Ω) and (*R*_s_ = 0.9 Ω and *R*_ct_ = 0.19 Ω) respectively. The ZIF-67 Co(OH)F@CO_3_O_4_200 electrode was characterized with a lower charge transfer resistance in the redox electrolyte the resistance of the electrode is quite small due to its large surface area tremella-like structure which incorporate high electrolyte. The small *R*_s_ and *R*_ct_, values indicate the high capacitance of ZIF-67Co(OH)F@Co_3_O_4_/CC200 there for the best electrochemical performance.

To evaluate the electrochemical stability and specific retention of ZIF-67Co(OH)F@Co_3_O_4_/CC200 electrode GCD plots were acquired for 5000 cycles at a constant current density of 10 A g^−1^ with the potential window −0.2 V to 0.6 V is shown in Fig. 7G. The stability curve of Co(OH)F@Co_3_O_4_/CC200 which exhibit great cyclic stability with capacitance retention of 90% after 5000 charges discharge cycles. The electrode maintained linear and symmetrical GCD curves in the last 10 cycles (inset in Fig. 7G) which show excellent cyclic stability. The cyclic stability curve of the Co(OH)F electrode with capacitance retention of 78% after 5000 GCD charge–discharge cycles is shown in Fig. S6 in ESI.† The summary of the charge–discharge characteristic ZIF-67Co(OH)F@Co_3_O_4_/CC200 electrode has high electrochemical stability and enhances their specific capacitance the remarkable *C*_F_ is due to its high surface area. The tremella-like structure is retained during electrochemical evaluations which provides stability and also prevent the accumulation of nanosheets.

**Fig. 8:**
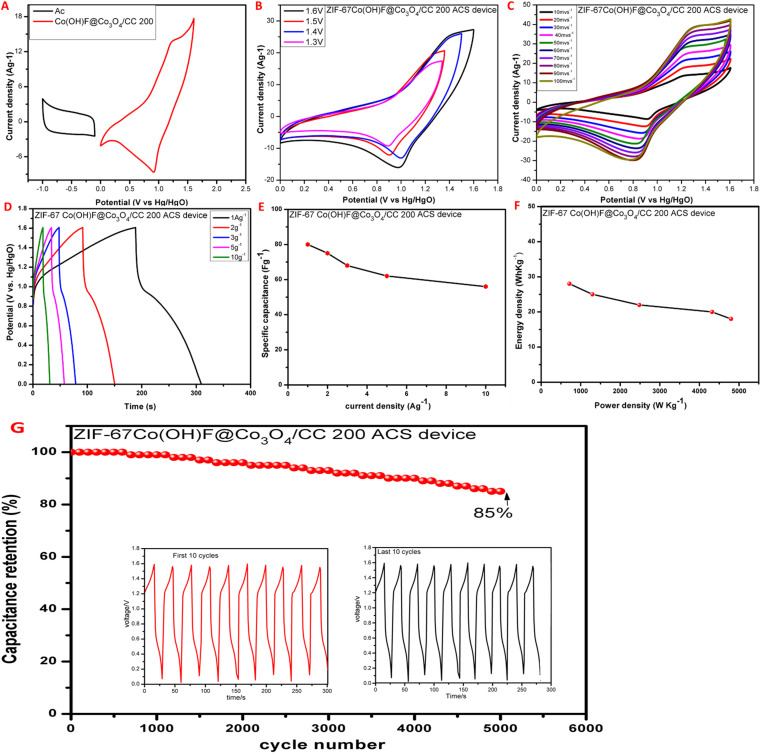
Electrochemical performance of ZIF-67Co(OH)F@Co_3_O_4_/CC200//AC ACS device, (A) CV curve of ZIF-67Co(OH)F@Co_3_O_4_/CC200 and AC carbon electrodes at 20 mV s^−1^, (B) CV curves of ZIF-67Co(OH)F@Co_3_O_4_/CC200//AC ASC device at 50 mV s^−1^ with different potential windows, (C) CV curve of ZIF-67Co(OH)F@Co_3_O_4_//AC ASC device at scan rates ranging from 10 mV s^−1^ to 100 mV s^−1^, (D) ZIF-67Co(OH)F@Co_3_O_4_/CC200//AC ASC device at current densities 1 A g^−1^, 2 A g^−1^, 3 A g^−1^, 5 A g^−1^, and 10 A g^−1^, (E) specific capacitance *versus* charge–discharge current of the ZIF-67Co(OH)F@Co_3_O_4_/CC200//AC ASC device, (F) Ragone plot showing the gravimetric energy density and power density of ZIF-67Co(OH)F@Co_3_O_4_/CC200//7AC ASC device, (G) cyclic stability at a current density of 10 A g^−1^, of ZIF-67Co(OH)F@Co_3_O_4_/CC200//AC ASC device.

To study the practical application of ZIF-67Co(OH)F@Co_3_O_4_200/CC the solid-state asymmetric supercapacitor (ASC) was constructed in a 1 M KOH solution. The separation voltage window for the activated carbon (AC) and ZIF-67Co(OH)F@Co_3_O_4_/CC200, electrodes is −1 to 0.1 V and 0–1.6 V, respectively shown in Fig. 8A. At the same time to determine the testing potential range of ASC, a series of cyclic voltammetry (CV) experiments were conducted within the potential range of 1.2 V, 1.3 V, 1.4 V, 1.5 V and 1.6 V with a constant scan rate of 50 mV s^−1^ as shown in Fig. 8B. The electrode materials can function in the potential window of 0 V to 1.6 V. As a result, potential range of 0 V–1.6 V was chosen. The CV curves are highly overlapped at all potential window confirming high reversibility at all condition. Fig. 8C display the CV curves of ASC device at different scan rates, ranging from 10 mV s^−1^ to 100 mV s^−1^. The peaks area of CV curves increases with increase the scan rate and the redox peaks are almost locate at similar potential. The galvanostatic charge–discharge (GCD) curves is shows in Fig. 8D. According to the GCD curve calculations, the ZIF-67Co(OH)F@Co_3_O_4_/CC200//AC ASC device shows the specific capacitance vary throughout current densities, 80 F g^−1^, 75 F g^−1^, 68 F g^−1^,62 F g^−1^ and 58 F g^−1^ at 1 A g^−1^, 2 A g^−1^, 3 A g^−1^, 5 A g^−1^, and 10 A g^−1^ respectively. The specific energy density and specific power density is shows in Fig. 8E and F, respectively. The electrode displayed maximum energy density of 28 W h kg^−1^ at a power density of 720 W kg^−1^. The energy density still remined of 18 W h kg^−1^ at a power density of 4800 W kg^−1^. Fig. 7G shows ASC device's cyclic stability and specific retention over 5000 cycles of GCD testing at 10 A g^−1^. After 5000 GCD cycles, revealing the excellent cycling stability of ASC device. The charging–discharging curves of the device at last 10 cycles (inset of Fig. 8G) are similar to those of the first 10 cycles, which indicates excellent cycling features the device maintained 85% of its capacitance, demonstrating good stability it is due to its high surface area tremella like structure.

We compare this work with the previous literature work it prove that this work has excellent cyclic stability which is shows in Table 1.

**Table 1:** Shows the comparison of specific capacitance of the ZIF-67-Co(OH)F@Co_3_O_4_ nanocomposite with related electrodes materials

Electrode materials	Electrolyte	Current density A g^−1^	Specific capacitance F g^−1^	Cyclic stability at 10 A g^−1^	Ref.
Pure Co_3_O_4_	3 M KOH	1	371	68.8%	55
Porous mixed phase CoO/Co_3_O_4_	3 M KOH	1	431	90%	56
CuO_2_/CuO/Co_3_O_4_ core shell nanowires	3 M KOH	0.5	318	90%	57
Core shell Co_3_O_4_ nano sphere	2 M KOH	0.5	216	80%	58
Co_3_O_4_/CoO@ carbon spike like grains	1 M KOH	1	324	88%	59
ZIF-67Co(OH)F@Co_3_O_4_/CC	1 M KOH	1	442	90%	This work

## 4. Conclusion

In this work ZIF-67Co(OH)F@Co_3_O_4_, metal organic framework was synthesized on carbon cloth by *in situ* technique followed by hydrothermal method using the template ZIF-67 precursor. The ZIF-67Co(OH)F@Co_3_O_4_/CC electrode show a satisfactory specific capacitance of 442 F g^−1^ at 1 A g^−1^ and superior capability with 90% retention with 10 A g^−1^ after 5000 charges discharge cycles. These advantages are due to its high surface area of tremella like structure and the synergetic interaction between ZIF-67Co(OH)F@Co_3_O_4_ and carbon cloth. The factor enhances the electro conductive and electroactive sites for faradaic redox reaction.

## Data availability

The data that support the findings of this study are available from the corresponding author upon reasonable request. Due to [state any restrictions, *e.g.*, privacy or ethical concerns], some data may not be publicly available.

## Conflicts of interest

The authors declare that they have no known competing financial interests or personal relationships that could have appeared to influence the work reported in this paper.

## Supplementary Material

RA-014-D4RA04250F-s001
