# Supplementary material for: A tremella-like in situ synthesis of ZIF-67Co(OH)F@Co3O4 on carbon cloth as an electrode material for supercapacitors
Source: RSC Adv. 2024 Sep 4;14(38):27831–42. doi: 10.1039/d4ra04250f (PMC11372565; doi:10.1039/d4ra04250f)
Supplement: RA-014-D4RA04250F-s001 [file RA-014-D4RA04250F-s001.pdf]

Supplementary Information (SI) for RSC Advances.
This journal is © The Royal Society of Chemistry 2024

Supporting information:

A *tremella-like Insitu* synthesis of ZIF-67Co(OH)F@Co_3O_4 upon carbon cloth as an electrode material for supercapacitor

Shakeel Ahmad[a], Muhammad Tariq[a], Zia Ur Rehman*[b], Shanshan Yao[b], Bing Zhu[a], Henmei Ni*[a], Muhammad Samiuddin[c], Khalid Ali Khan[d], Magdi E.A. Zaki[e]

a. *School of Chemistry and Chemical Engineering, Southeast University, Nanjing 211189, PR China*
b. *1Institute for Advanced Materials, College of Materials Science and Engineering, Jiangsu University, Zhenjiang 212013, P. R. China*
c. *Metallurgical Engineering Department, NED University of Engineering and Technology, Karachi, 75850, Pakistan,*
d. *Advanced Materials Science (RCAMS), King Khalid University, Abha 61413, Saudi Arabia*
e. *Department of Chemistry, Faculty of Science, Imam Mohammad Ibn Saud Islamic University, Riyadh 13318, Saudi Arabia*

Corresponding author: Henmei Ni (*henmei_ni@seu.edu.cn)

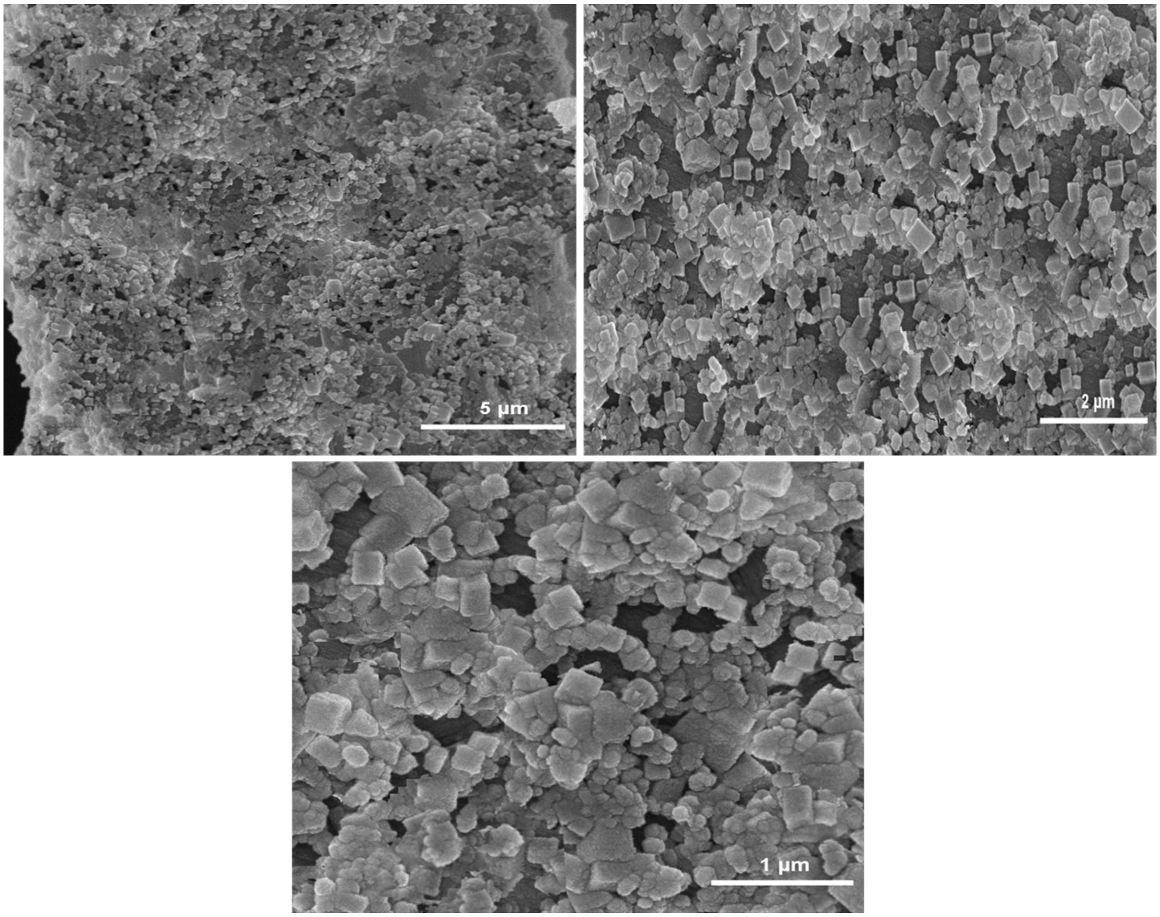

Fig S1. SEM images of ZIF-67Co(OH)F/ CC120 at different magnification.

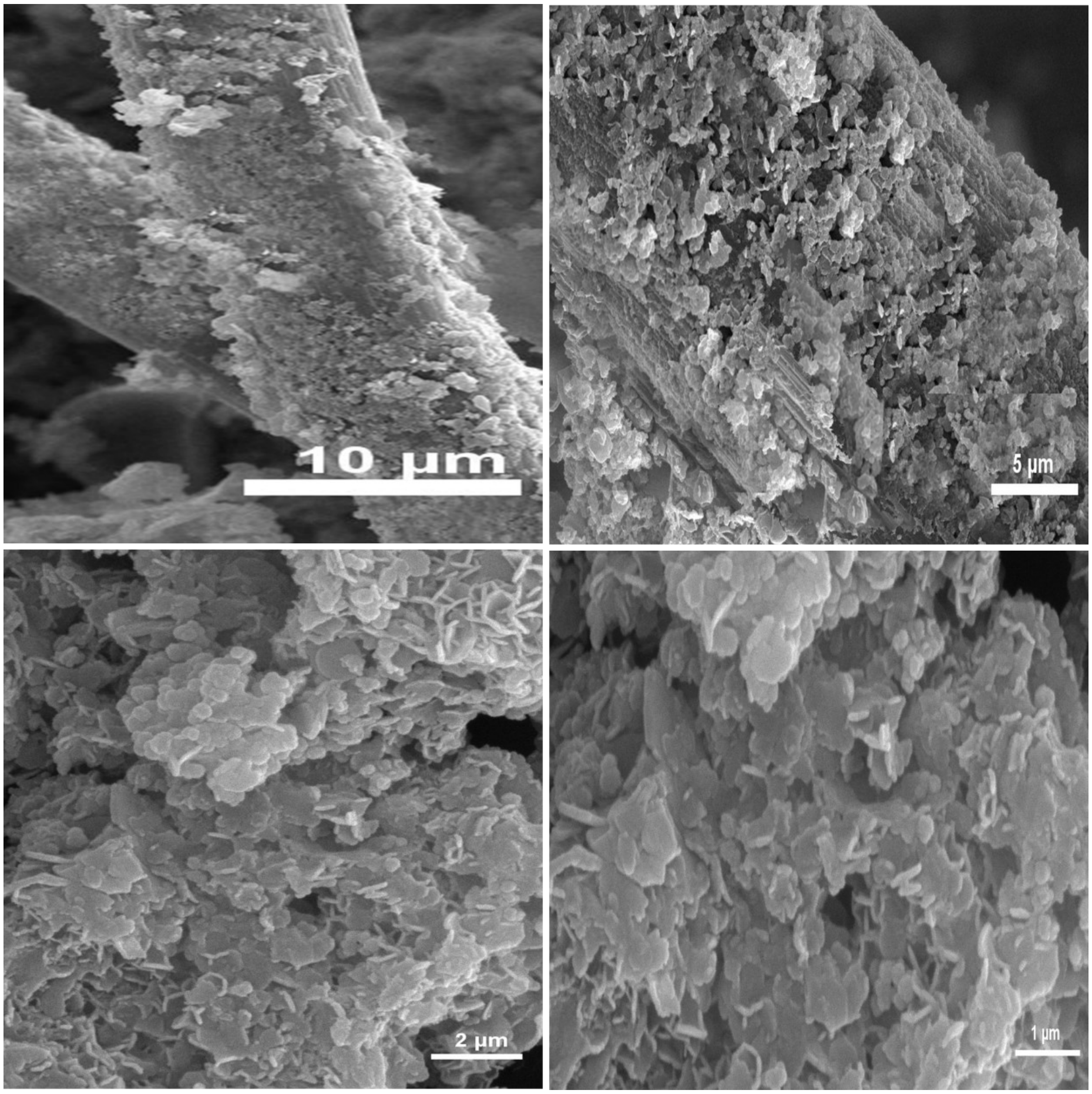

Fig S2. SEM images of ZIF-67Co(OH)F@Co_3O_4/ CC 160 at different magnification.

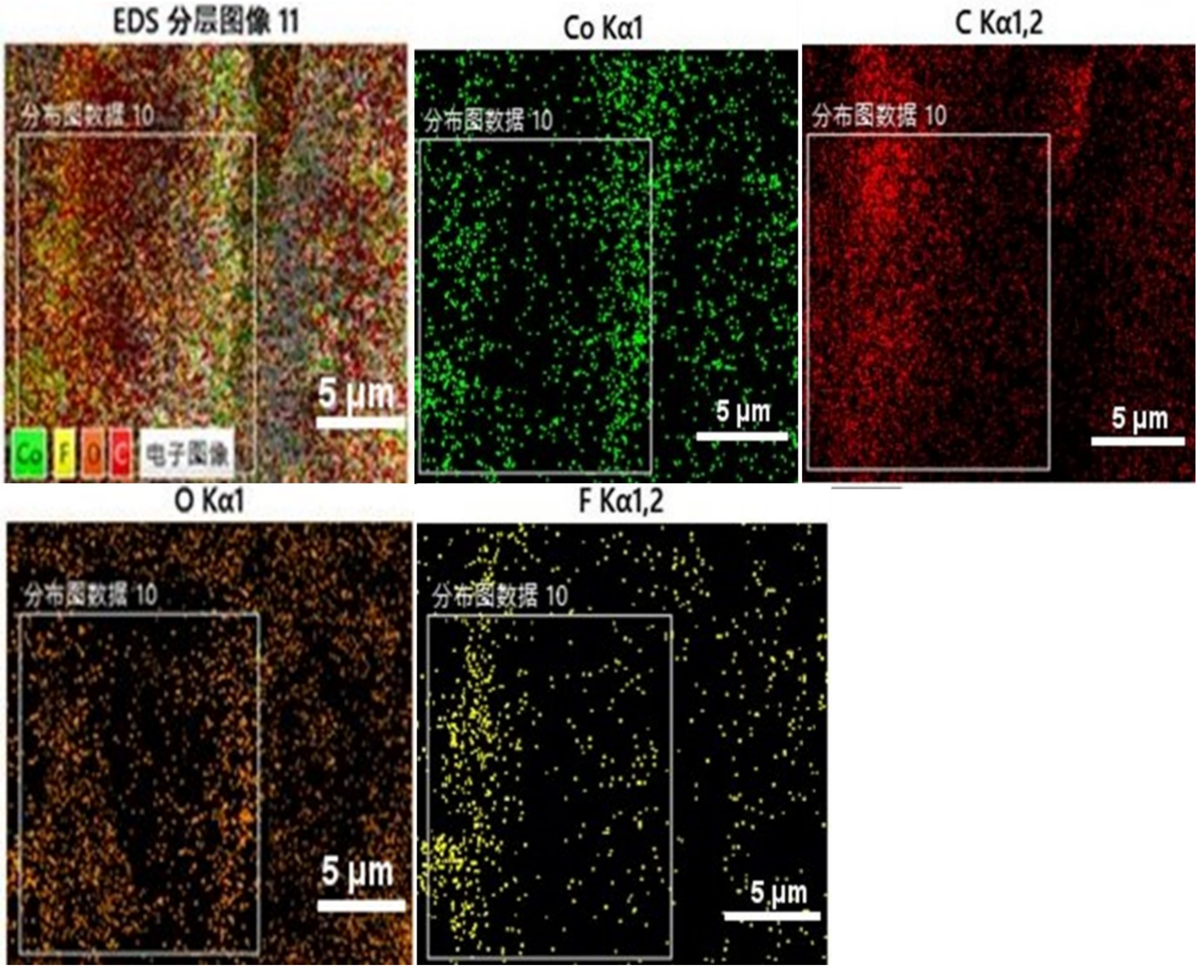

Fig S3. EDS mapping result of O,C,Co, and F in ZIF-67Co(OH)F@$Co_3O_4$200

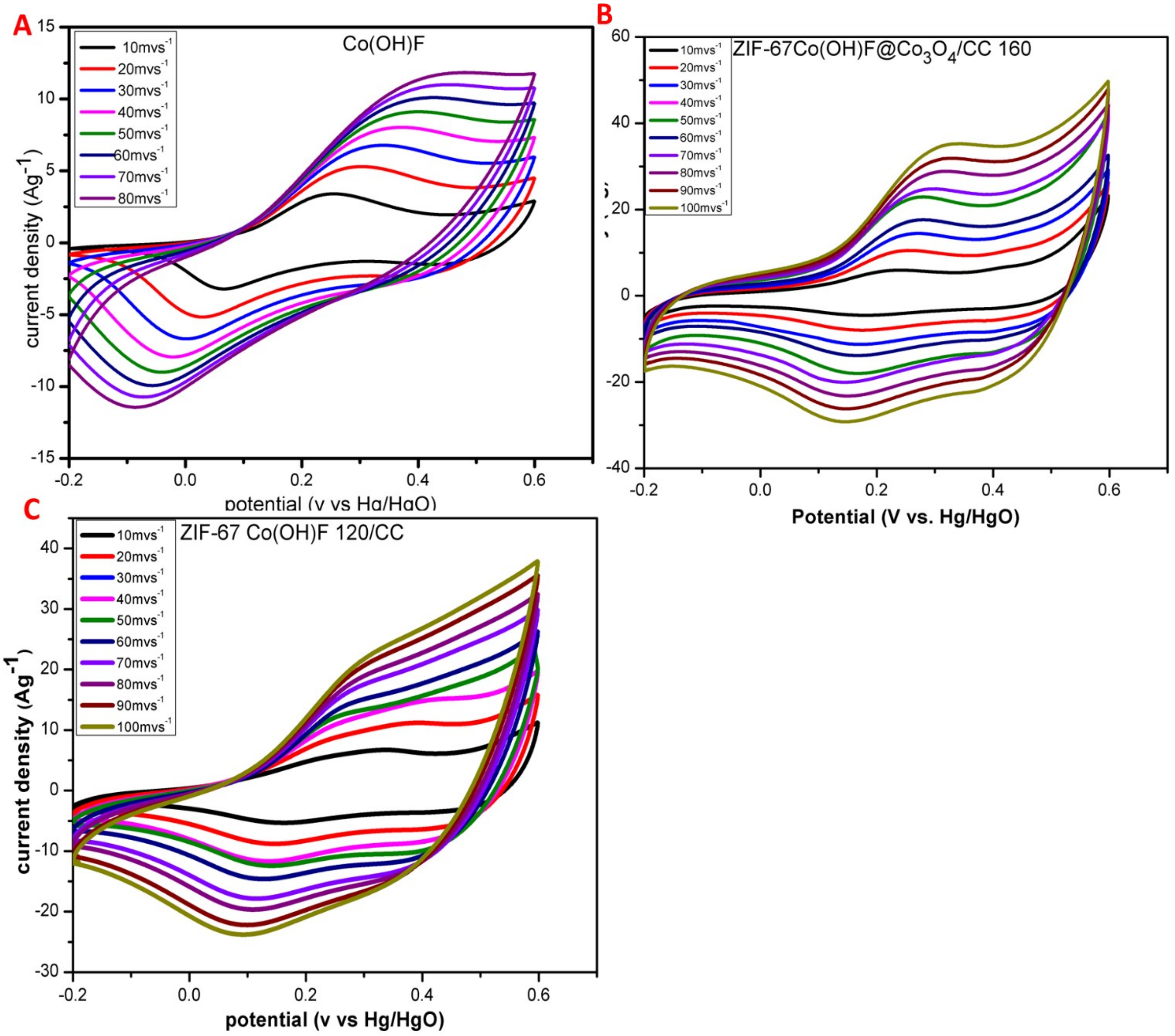

Fig. S4. (a) CV carves of Co(OH)F (b) CV carves of ZIF67-Co(OH)F@$Co_3O_4$160(c) CV carves of ZIF67-Co(OH)F120

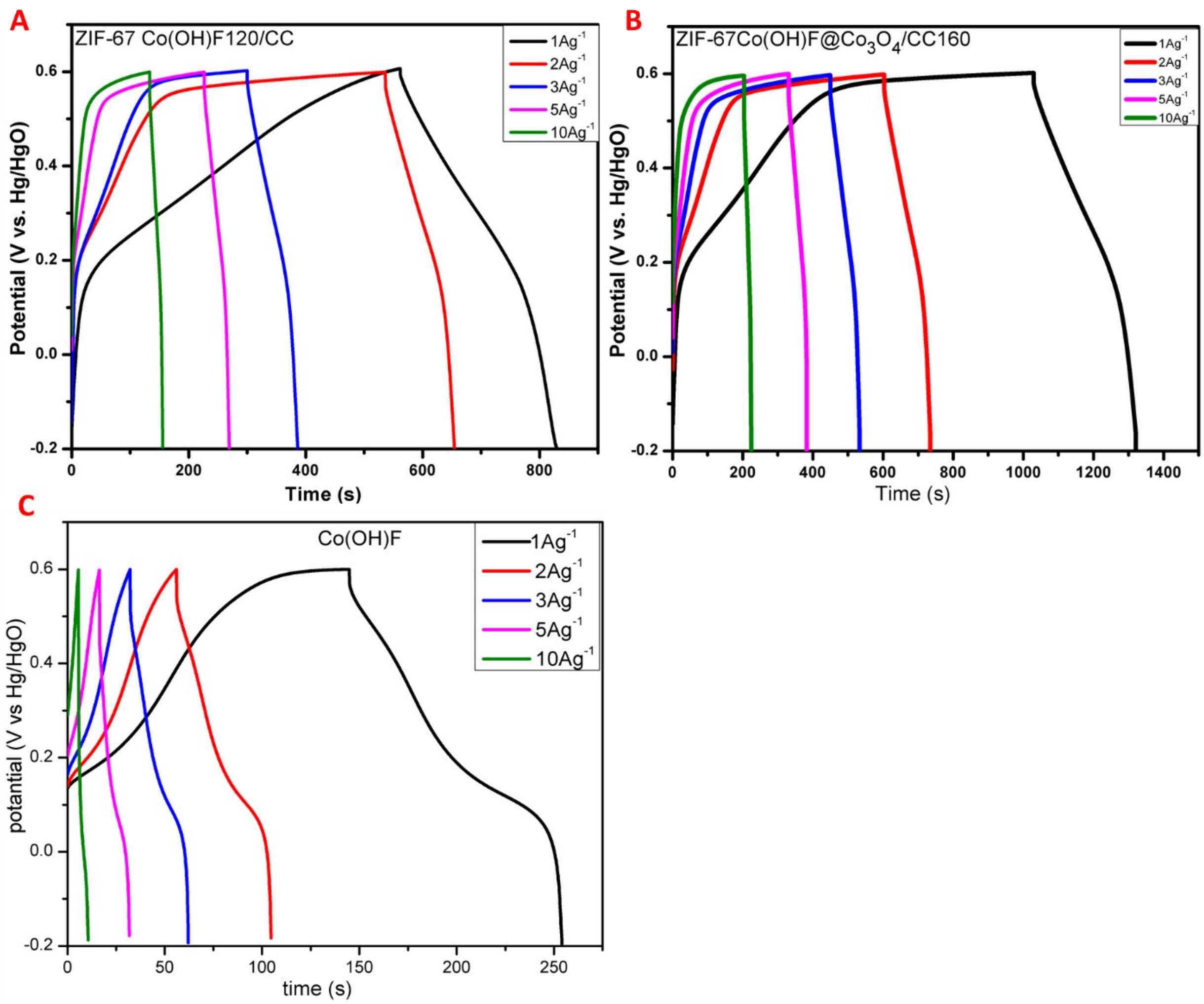

Fig. S5. (a) GCD carves of ZIF67-Co(OH)F/CC 120, (b) GCD carves ZIF67-Co(OH)F@$Co_3O_4$160(c)) GCD carves of Co(OH)F

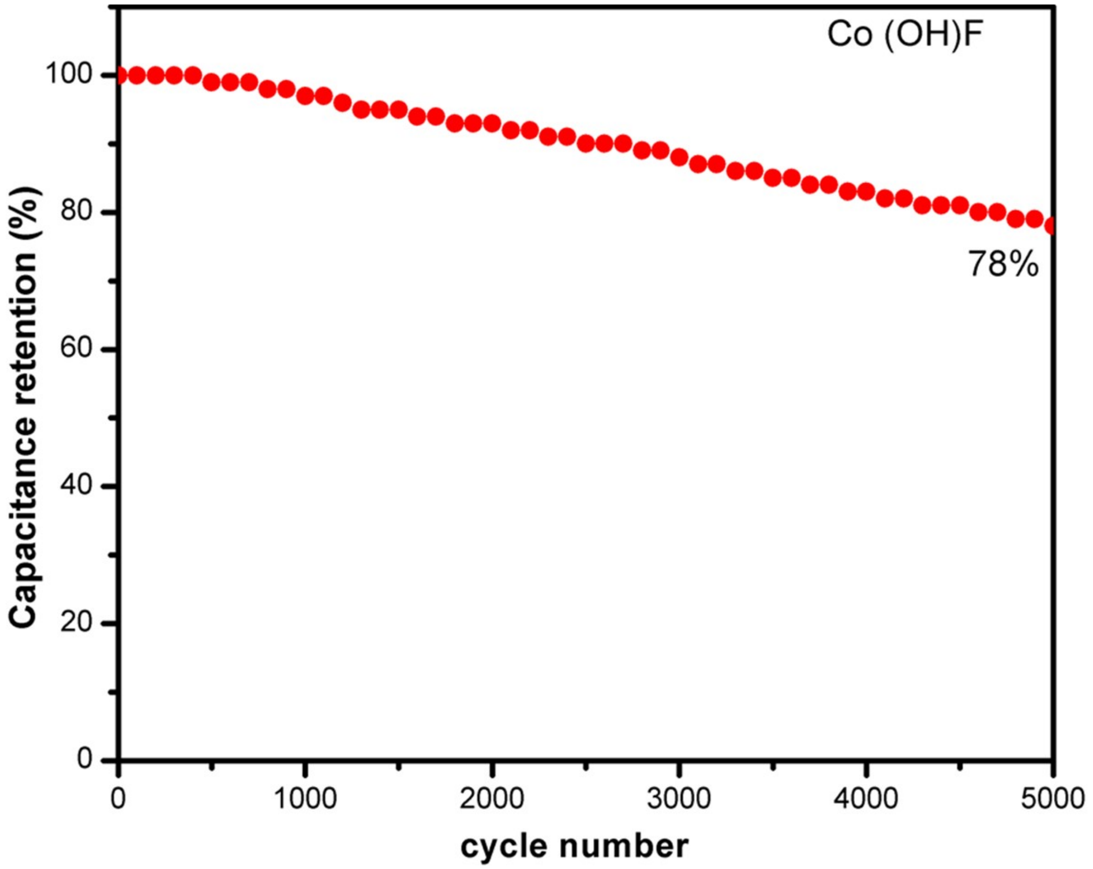

Fig.S6. cyclic stability at current density of $10Ag^{-1}$ of Co(OH)F electrode.
